# Effects of Chloroform on the Blood

**Published:** 1853-07

**Authors:** 


### ARTICLE XIII.

### *Effects of Chloroform on the Blood.*

Mr. D. Chaumont read before the Edinburgh Physiological Society the following communication:

Much controversy has arisen as to the real nature of the effect of chloroform on the blood. Amussat, Sédillot, etc., have contended that the blood is darkened by it, and that the effect is quite analogous to asphyxia. Gruby, on the other hand, contends that not only is the arterial blood not darkened but that the venous blood is even rendered as bright as the arterial. I have found that when blood is agitated with chloroform it does not coagulate, but becomes quite transparent; and when the blood is venous the color is rendered as brilliant as that of arterial blood. Sulphuric ether, on the contrary, does not affect the color of venous blood. Chloroform seems thus to have a special effect of brightening colors, not only in this but in other cases; as, for instance, when it dissolves iodine, the solution has not the deep-brown color obtained when water, alcohol, &c., are used, but has the beautiful violet hue of the vapor. In some cases chloroform restores the color of old blood-stains on linen, &c., but the effect is not constant. When blood, acted upon by that agent, is examined by means of the microscope, the red globules are found completely dissolved, the field presenting only a homogeneous colored expanse, in which the white globules float unaffected. Chloroform acts on pus very similarly to acetic acid, as it brings into view the double and tripartite nuclei of the globules, although the effect is not so speadily accomplished as by acetic acid. When the blood of leucocythemia is acted upon, the red globules dissolve, and the white present the reaction not of the ordinary white globules of the blood but of pus. With the view of observing the effects of the administration of chloroform on the blood in the living body, I attempted to anæsthetize a rabbit by the rectum, so as not to interfere with the respiration. Although two drachms were injected, no other effect was produced except an intoxicated appearance, which did not give immunity from pain. This corresponds with what was observed by Flourens in the early days of etherization. Another rabbit was anæthetized by inhalation in the ordinary way, and died suddenly during the operation. On opening the body the lungs we found florid, and without trace of asphyxia; the auricles of the heart continued to contract for an hour after death; the peristaltic action of the intestines and bladder continued also with tolerable vigor. Venous blood from this animal presented the same reactions as before mentioned. The next experiment was upon mice. Four of these animals were placed alive in a wide-mouthed bottle, and a few drops of chloroform poured into the bottom of the vessel. This speedily caused a great excitement among them, and they soon after died. The examination of their bodies showed the same signs as in the rabbit. The brain was also normal. Another rabbit was anæsthetized by the lungs, and the crural vessels of the right leg exposed and carefully watched during the whole process of inhalation, but without there being any evidence of change in the color of the venous or the arterial blood. In this animal also an attempt was afterwards made to induce insensibility by the rectum, but without success. The chloroform was in this case mixed with water, and the intoxicating effect produced was greater and continued longer than in the former. The temperature was also apparently lowered, although no exact observation by the thermometer was made. Duméril and Dumarquay have stated that the temperature is lowered in anæsthesia, and under the influence of ether and alcohol, distinguishing the effects of these agents from common narcotism, as by laudanum, in which the temperatúre is raised. This last rabbit died after a few hours, and the *post-mortem* appearances showed that death had been occasioned by acute peritonitis. Here too, there were no signs of asphyxia, the lungs being of a bright rose color. In the liver was found a cyst, containing a white putty-like mass, which, under the microscope, showed beautiful oval-shaped cells containing a round nucleus, with several, generally three, oval nucleoli. These, Dr. Bennett informed me, were entozoa. The action of chloroform on them was curious; the nucleus swelled up so as to fill the cell, while in another part of the field were seen cells floating empty, and nucleoli floating about in threes, joined by filaments. Hence, it seemed as if the nucleus had alone been dissolved, a curious fact when taken in connection with the solution of the red globules of the blood, these being also considered as nuclei.

The above experiments were all subsequently confirmed by a committee of the Royal Medical Society, by whom it was also observed that chloroform dropped upon the intestines and bladder caused violent peristaltic action, and that dropped upon the heart, while that organ was contracting, it caused a cessation of the pulsations until it had all evaporated, having a temporary paralyzing effect on that organ.

From the above experiments I am inclined to think that chloroform does not act by asphyxia, and that, when this does occur, it results from the manner of the administration; that its action is directly upon the nervous centres, first upon that part connected with the perception of sensation, and lastly on the spinal cord, or generator of motive force; that although it arterializes the color of the venous blood out of the body, I have not yet been able to observe the same effect in the body; and that, on the other hand, the arterial blood is in no way affected in color by its inhalation.

Dr. Bennett referred to a report on the action of chloroform drawn up by himself, and printed in the *Transactions* of the Edinburgh Medico-Chirurgical Society about the time when chloroform was introduced as an anæsthetic. (*Monthly Journal*, Jan. 1848.) The results of the experiments there detailed were similar in some respects to those of Mr. De Chaumont.

Dr. Matthews Duncan remarked, that rabbits were not good subjects for experiments with chloroform, on account of the rapidity with which fatal effects succeeded the anæsthesia. From recent trials, he was convinced that chloroform introduced into the stomach could produce anæsthetic effects.—*Monthly Journal of Medical Science*, May, 1853.

